# A high-resolution dataset of water bodies distribution over the Tibetan Plateau

**DOI:** 10.1038/s41597-024-03290-4

**Published:** 2024-05-04

**Authors:** Zhengchao Chen, Linan Guo, Yanhong Wu, Bing Zhang, Pan Chen, Xuan Yang, Jiawei Guo

**Affiliations:** 1 State Key Laboratory of Remote Sensing Science, Aerospace Information Research Institute, Chinese Academy of Sciences, Beijing, 100094 China; 2 International Research Center of Big Data for Sustainable Development Goals, Beijing, 100094 China; 3 China University of Mining & Technology-Beijing, Beijing, 100083 China; 4 Key Laboratory of Digital Earth Science, Aerospace Information Research Institute, Chinese Academy of Sciences, Beijing, 100094 China; 5 Aerospace Information Research Institute, Chinese Academy of Sciences, Beijing, 100094 China; 6 University of the Chinese Academy of Sciences, Beijing, 100049 China; 7 Center for Geo-Spatial Information, Shenzhen Institutes of Advanced Technology, Chinese Academy of Sciences, Shenzhen, 518055 China; 8 China Remote Sensing Satellite Ground Station, Aerospace Information Research Institute, Chinese Academy of Sciences, Beijing, 100094 China

**Keywords:** Hydrology, Freshwater ecology

## Abstract

Water body (WB) extraction is the basic work of water resources management. Tibetan Plateau is one of the largest alpine lake systems in the world. However, research on the characteristics of water bodies (WBs) is mainly focused on large and medium WBs due to spatial resolution. This research presents a dataset containing a 2-m resolution map of WBs in 2020 based on Gaofen-1 data, and morphometric and landscape indices of WBs across the Tibetan Plateau. The Swin-UNet model is well performed with overall accuracy at 98%. The total area of WBs is 56354.6 km^2^ across Tibetan Plateau in 2020. The abundance compared with that from size-abundance relationship indicate WBs in the Tibetan Plateau conformed to the classic power scaling law. We evaluate the influence of spatial-resolution in WB extraction, which shows the dataset could be valuable to fill the gap of existing WBs map, especially for small waters. The dataset is valuable for revealing the spatial patterns of WBs, and understanding the impacts of climate change on water resources in Plateau.

## Background & Summary

Terrestrial water bodies (WBs), such as lakes, ponds, and reservoirs, are essential components of the hydrological and biogeochemical water cycles^1^, which provide essential ecosystem services for human society, such as river flow, biodiverse habitats, fisheries, and supplying irrigation water^2,3^. Monitoring the dynamic changes of WBs provides important information on understanding changes of the surrounding regions^2,4^.

Understanding the abundance and size distribution of global or regional WBs has been a persistent effort for several years. Traditionally, this information comes from map compilations^1^ and statistical extrapolations based on abundance-size relationships^5–7^. However, map compilation tends to underrepresents small WBs^8^, while statistical extrapolations of abundance likely overestimate abundance of small WBs^9–11^. The morphology of WBs can quantitatively describe the geometric features of water landscapes, such as water area, depth, shoreline length, shoreline development index^12^. Morphological characteristics of WBs influence the ecological functionality in a region and is hard to be obtained. Benefiting from the comprehensive information of high-resolution satellite imagery, it is possible to accurately resolve the abundance, size distribution and morphological characteristics of WBs at large scale^13,14^.

Water body extraction is the basic work of water resources management^15^. The extraction of WBs in large scale from remote sensing images can be considered as a target detection process^16^, which mainly includes single-band density slicing^17^, spectral water indexes^18,19^, object-oriented approaches, and deep learning methods^20^. There are several researches for regional or global WBs extraction in past decades. The global surface water dataset (GSWD) developed by Pekel *et al*.^21^ describes the changes of WBs from 1984 at 30 m resolution^21^ and updated to 2022 now. Verpoorter *et al*.^5^ inventoried the world’s lakes larger than 0.002 km2 in size, including the information of abundance, size (i.e., area and perimeter), geographical distribution, elevation, and morphometric characteristics such as the shoreline development index (SDI)^5^. Spatial and temporal changes of inland WBs in China were investigate by Ma *et al*.^2^ and Zhang *et al*.^22^, respectively. Feng *et al*.^23^ used the GSWD dataset to report that previous studies^24^ underestimate the abundance and area of WBs (>1 km2) in China. Besides surface water bodies, dataset of large dams and reservoirs was also generated by Wang *et al*.^25^.

**Fig. 1:**
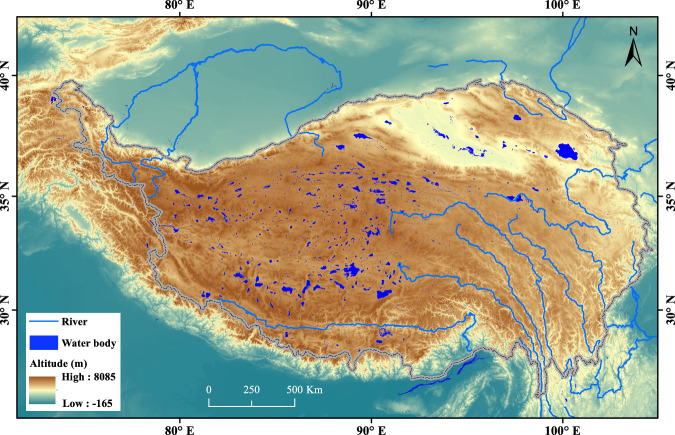
Study area.

Although the overall global patterns of water body changes have been analysed, regional analyses are sorely needed, especially for small waters at regions that are sensitive to climate change. Recently, deep learning received widespread attention for water bodies recognition. Compared to the traditional machine learning methods, deep learning relies heavily on large-scale training data^26^. Transfer learning is an emerging method that is applicable when the training data is limited. Fine-tuning a pretrained CNN (Convolutional Neural Network) model may be an effective strategy for many deep learning model applications. At present, high-resolution water body extraction based on deep learning method is mainly implemented at local-scale^27,28^. One study from Fang *et al*.^16^ extracted man-made reservoirs from Landsat-8 images based on a CNN model, ResNet-50 globally^16^. Tibetan Plateau (Fig. 1), on where are more than 1,100 alpine lakes^29^ with area larger than 1 km^2^, receive much attentions because of its less effect by human activities. At present, the extraction of water bodies mainly considers lakes larger than 1 km^2^ on the Tibetan Plateau^23^. However, due to the melting of glaciers, the abundance of small water bodies on the Tibetan Plateau will continue to increase^30^, which is still unknown for us. It is better to obtained this information rely on very high spatial resolution remote sensing images. In addition, although there are several medium and high-resolution images, it is still unclear in data source selection with appropriate resolution for different objectives.

In this study, a 2m-resolution map of water bodies on the Tibetan Plateau is produced based on visual transformer model from Gaofen-1 data. Morphological and landscape indices of WBs are included in the dataset. At the same time, we compare the WBs extraction from different resolutions, which helps analyze the influence of spatial resolution on extraction of water body at different size. The dataset could be valuable for accessing the spatial patterns of WBs, testing the validity of controversial power scaling law for the size-abundance relationship, and selecting data source for water body extraction on the Tibetan Plateau.

## Methods

### Data

Gaofen-1 (GF-1) is the first of the Gaofen series satellites, which was launched on April 26, 2013. The GF-1 satellite is equipped with one 2-m-resolution panchromatic sensor and one 8-m-resolution multispectral sensor. It also has four 16-m-resolution wide-field-of-view (WFV) multispectral sensors. The GF-1 satellite is suitable for surface water distribution analyse. panchromatic and multi-spectral images in 2020 were used in this study. Before water extraction, we used the pansharp fusion method to fuse the panchromatic images and multi-spectral images to generate the images with a spatial resolution of 2 m and four bands.

### Water body extraction based on deep learning

**Fig. 2:**
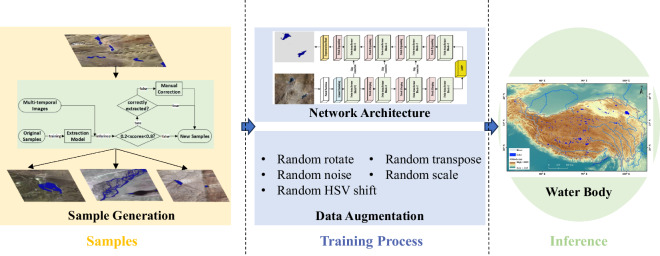
The flowchart of water body extraction algorithm.

To extract water bodies precisely over a wide range and multiple time periods, this study trained Swin-UNet^31^ network based on samples from rapid sample generation technique, combining numerous data augmentation strategies, ultimately achieving the recognition of water bodies over the Tibetan Plateau. The process of extraction algorithm is shown as Fig. 2.

As a data-driven algorithm, the performance of deep learning algorithms is greatly influenced by the quality and quantity of samples. In this study, we designed a rapid sample generation method based on semi-supervised principles (left part in Fig. 2). This process began with a small number of manually labelled samples to train a simple water body recognition network (approximately 800 samples in the size of 512 × 512, in which 100 samples contained water bodies). A threshold-based approach, supplemented by manual inspection and correction, was employed to quickly clean the inference results. The cleaned inference results were then reintegrated into the training samples as the new samples. In the process, areas with confidence scores greater than 0.8 or less than 0.2 were considered correctly classified, while misclassifications were inspected and corrected manually. After several rounds of sample generation, totally around 6000 samples in the size of 512 × 512 were obtained, comprising 778 samples containing water bodies and the rest being totally backgrounds. These samples were divided into training, validation, and test sets in a ratio of 7:1:2.

Because of large-scale variations and strong spatial continuity of water bodies, Swin-UNet network is suitable in this study with large receptive field and strong spatial modelling capabilities. Additionally, multi-spectral information encoding was introduced to make full use of the spatial information and spectral information of water body. In order to make full use of deep semantic information while preserving spatial information, the features from the encoder and decoder interacted through skip connections^32^. In the bridge between the encoder and decoder, an Atrous Spatial Pyramid Pooling (ASPP) module^33^ was introduced to further extract image texture features under multiple receptive fields. In the encoding phase, the input image was encoded through Patch Partition and Linear Embedding. In the decoding phase, the multi-scale features were decoded through multiple Swin-transformer Blocks and Patch Expanding. In the Swin-transformer Block, the input features were first normalized by Layer Normalization (LN). Then, shifted window-based multi-self-attention (SW-MSA) was used to model global features within a window. Subsequently, the outputs of SW-MSA were added to the input features and normalized again with LN. Finally, the normalized features were fed into a simple Multilayer Perceptron (MLP). In the study, all Swin-transformer Blocks were used in pairs, utilizing two stacked SW-MSA with shifted windows to capture the global receptive field across the entire image.

To enhance the model’s adaptability to images captured under different regions and imaging conditions in the inference process, several data augmentation strategies were applied during the training process. Including: (1) Random HSV (Hue, Saturation, Value) jittering: Randomly converting the image to the HSV color space and adding jittering (−30 < H < 30, −15 < S < 15, −30 < V < 30) to simulate a broader range of color variations; (2) Random Gaussian noise addition: Randomly adding Gaussian noise to the sample images to simulate images with different levels of noise (mean value = 0 and variance <50); (3) Random rotation: Randomly rotating the images by a certain angle (−180° to 180°) to simulate observations of objects from different directions; (4) Random scaling: Randomly scaling the images by a certain factor (0.9 to 1.1) to simulate variations in image quality and sharpness under different shooting conditions; (5) Random flipping: Randomly performing horizontal (left-right) mirror flips on the sample data to simulate image data with different spatial arrangements of objects. All the data augmentation algorithms used in this study were implemented based on the open-source library “albumentations”^34^. All data augmentation functions have a probability of 50% to be applied.

### Indices to evaluate spatial variation of water bodies

Besides water abundance and water area, morphometric indices including the shoreline perimeters (SP), and shoreline development index (SDI) were calculated based on high-resolution water body map. The SDI reflects the degree of shoreline irregularity. The more irregular of shoreline, the more habitat diversity the lake can provide for the coastal zone (SDI = 1 when the water body is circle). In addition, Water bodies are important landscape. Here we obtained the landscape pattern indices to understand the geographical significance of water bodies morphological characteristics and distribution rules. Overall, 9 indices were considered in this study (Table 1).

**Table 1:** Indices metrics.

Indices	Full name	Description	Equation
Morphometric indices	Abundance	Number of water bodies	
	Area	Area of water body	
	Shoreline perimeters	Shoreline perimeter of water body	
	Shoreline development index	Reflects the degree of shoreline irregularity	$SDI=\frac{SP}{2\sqrt{\pi A}}$
Landscape indices	Patch density	Number of patches per unit area	$PD=\frac{{n}_{i}}{A}\left(1000000\right)\left(100\right)$
	Largest patch index	The percent of the total landscape that is made up by the largest patch	$LPI=\frac{\max ({a}_{ij})}{A}\times 100$
	Landscape shape index	The complexity of the shape	$LSI=\frac{0.25\ast {\sum }_{j=1}^{n}{P}_{ij}}{\sqrt{A}}$
	Splitting index	The number of patches if all patches the landscape would be divided into equally sized patches	$SPLIT=\frac{{A}^{2}}{{\sum }_{j=1}^{n}{a}_{ij}^{2}}$

## Data Records

The map and statistic indices data of inland water bodies across Tibetan Plateau in 2020 is archived and openly accessible at Figshare^35^ via the link: 10.6084/m9.figshare.24616491.v2. Table 2 shows the details of dataset. 2675 tiles (GeoTIFF format, 16784 × 16784 pixels) are compressed into the Tibet_water_2020_2m.rar file, which is the water bodies distribution with 2-m spatial resolution across Tibetan Plateau in 2020. The value is 11 for pixels classified as water. The file name of the tiles referred to the Google zoom level. Description about these tiles is shown in the TIFFlist.csv, including the longitude and latitude of top-left corner of each tile. The statistic results of water abundance, water area, and morphometric indices are shown in Indices.csv file, while four landscape indices (patch density, largest patch index, landscape shape index, and splitting index) are given in Landscape.csv file.

**Table 2:** Files and formats of the dataset.

File name	Description	
Tibet_water_2020_2m.rar	Map of water bodies across Tibetan Plateau in 2020 with 2-m resolution, including 2675 tiles (GeoTIFF, 16784×16784 pixels). Pixel value: 11-water, 0-others	
TIFFlist.csv	Longitude and latitude of top-left corner for each tile	
Indices.csv	Abundance	Water abundance of different size of water bodies
	Water area	Water area of different size of water bodies
	Shoreline perimeter	Perimeter of different size of water bodies
	Shoreline development index (SDI)	Reflects the degree of shoreline irregularity of different size of water bodies
Landscape.csv	PD	Patch density
	LPI	Largest patch index
	LSI	Landscape shape index
	SPLIT	Splitting index

## Technical Validation

### Quality control

The dataset was produced with strict quality control. To ensure the quality of samples, we inspected the misclassification water bodies manually in the rapid sample generation process. The Swin-UNet model is good performance which is evaluated by 5 indices (Table 3). OA, P and R could assess the precise of water extraction; F1 score is a comprehensive evaluation of the performance of water detection model; IOU is used to reflect the overlap of the truth and prediction region. TP (True positives) indicates pixel number that correctly detect water, FP (False positives) is the pixel number that incorrectly identified as water, TN (True negatives) indicates the pixel number correctly identified as non-water, while FN (false negatives) is the pixel number that incorrectly identified as non-water. Results (Table 3) show that the water extraction algorithm is an accurate method to detect water bodies in high-resolution remote sensing images with overall accuracy at 98%. The IOU is relatively low with 68%, which may result from the small covering proportion of water bodies in an image.

**Table 3:** Precision of SwinUNet model.

Index	Formula	Value
Overall Accuracy (OA)	$OA=\frac{TP+TN}{TP+TN+FP+FN}\times 100 \%$	98%
Precision (P)	$P=\frac{TP}{TP+FP}\times 100 \%$	82%
Recall (R)	$R=\frac{TP}{TP+FN}\times 100 \%$	79%
F1 score	$F1\;score=2\times \frac{\left(P\times R\right)}{\left(P+R\right)}\times 100 \%$	81%
Intersection over Union (IOU)	$IOU=\frac{TP}{TP+FP+FN}\times 100 \%$	68%

Then the extracted water bodies have been manually corrected based on visual interpretation. Before indices calculation, morphological opening-and-closing operation was employed. We first filled the small holes inside the water using closing operation to ensure the integrity of the target area, and then remove isolated small pixels using opening operation to ensure the minimal noise of the image. Ellipsoidal area and perimeter instead of Projection one should be used in QGIS software to ensure the correct statistic results. Due to the large study area and limited satellite passing time, a lot of winter images were used in the study, resulting in the extracted water area and abundance being smaller than that from wet season. The water distribution dataset has been compared also with other dataset with lower spatial resolution as described in detail below.

### Comparison of morphometric indices of WBs

There is little *in-situ* observation of number or area of WBs in large scale. Conventionally, WBs over large areas are characterized using 1 or a few snapshots of remotely sensed images. We herein compare our morphometric indices dataset with existing research. In our estimation, the abundance of water bodies (>0.01 km^2^) in the Tibetan Plateau is 96369, and the total area of these water bodies is 56354.6 km^2^(Table S1 in Supplementary). The total area of the WBs larger than 1 km^2^ is 51034.6 km^2^. According to Zhang *et al*.^36^, until 2018, there are 1424 lakes larger than 1 km^2^ in the Tibetan Plateau with total area of 5 × 10^4^ ± 791.4 km^2^. In addition, our estimation of the WBs larger than 1 km^2^ in the Tibetan Plateau is much higher than Mao *et al*. (46,264.5 km^2^)^37^ or Wan *et al*. (41,831 km^2^)^38^. This differences also demonstrate the results from previous studies^36,39^ that the WBs is expanding in the Tibetan Plateau.

**Fig. 3:**
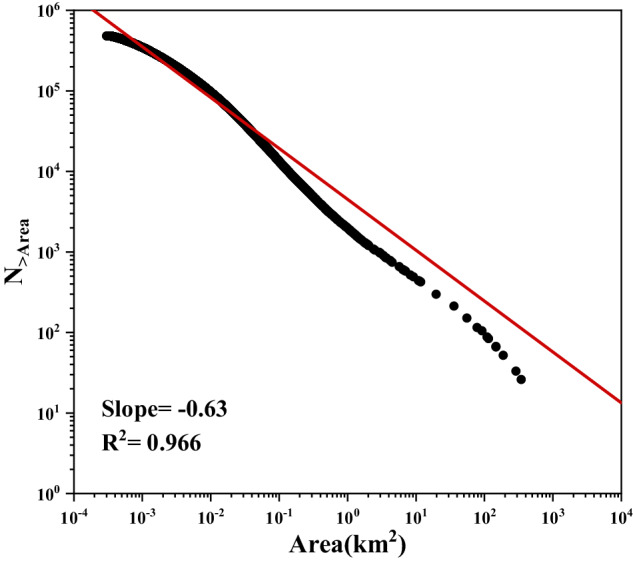
Log-abundance log-size plot of Water body size distribution.

Morphometric and landscape indices are included in our dataset. The statistic results are shown in Table S1, Table S2 and Figure S1 in Supplementary suggesting that small WBs (<1 km^2^) account for a large proportion of WBs in the Tibetan Plateau and are more separated. However, due to the limitation of water extraction algorithms and spatial resolution of remote sensing data, previous research is still lack in understanding the morphometric and landscape characteristics of small WBs. The size-abundance relationships were used to estimate the amount of WBs in large scale^10,40,41^. The size-abundance relationships conform to the power law^10^ based on the Pareto distribution probability density function.

1 $$N=c\times {A}^{-b}$$

where *N* is the number of water bodies greater than or equal to the area *A*, *c* is a constant.$b=D/2$, where *D* is the fractal dimension of the shorelines surrounding the water body area and is constrained between *D* = 1 (a population of perfectly smooth shorelines) and *D* = 2 (a population of shorelines so irregular they are space filling). The fractal dimension of size-abundance is supposed to be similar to the shoreline fractal dimension derived from dimensional analysis^23^. For WBs on the Tibetan Plateau, *D* is 1.263 (with R^2^ = 0.966), and Fig. 3 suggests that distribution deviates slightly from a true power law at WBs with larger area.

### Comparison of Water body extraction in different spatial-resolution

We further compared the dataset against two existing datasets with spatial resolution at 30 m and 10 m, respectively. The comparison analysis aims not only at the validation for our dataset, but to analyze the influence of spatial resolution and select applicable data source for water body extraction on the Tibetan Plateau. The European Space Agency (ESA) WorldCover is a land cover map that provides a new baseline global land cover product at 10 m resolution based on Sentinel-1 and 2 data that was developed and validated in almost near-real time and at the same time maximizes the impact and uptake for the end users. The Global Surface Water Explorer (GSWE) dataset was developed by the European Commission’s Joint Research Centre based on Landsat satellite images at 30-metre resolution^21^. The dataset maps the location and temporal distribution of water surfaces at the global scale during 1984 to 2022 at monthly and yearly, and provides statistics on the extent and change of those water surfaces.

**Fig. 4:**
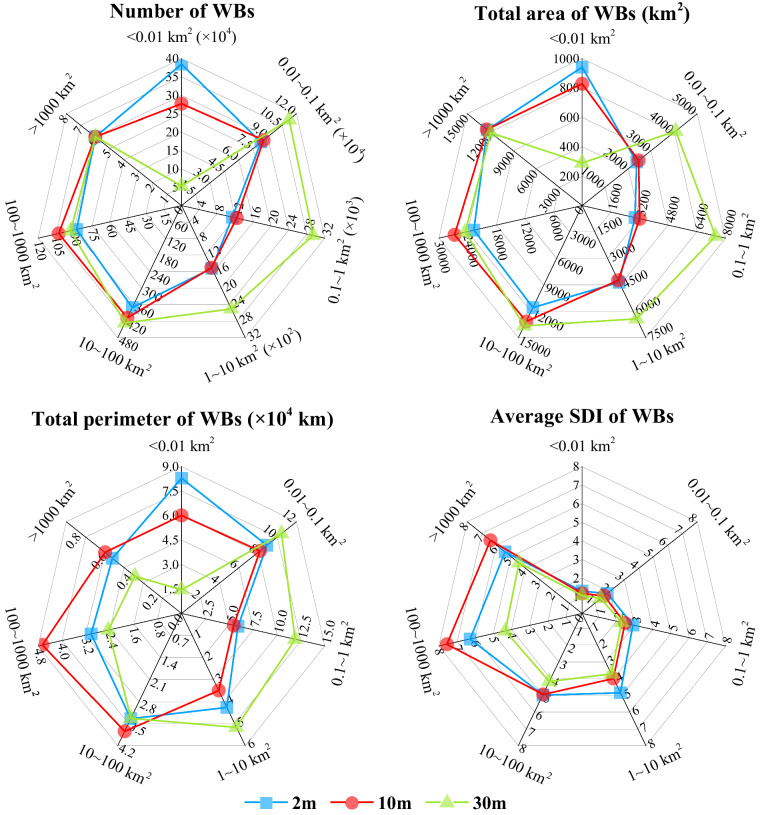
Water bodies distribution and morphometric characters based on different spatial-resolution.

Figure 4 shows the comparison of morphological indices extracted at different resolutions. For water bodies larger than 1000 km^2^, the estimated number and area of WBs from data at different resolutions are similar. However, there is a significant inconsistency in perimeter estimation for large water bodies, which may result from the influence of coarse resolution of 30-m data. There are limitations to estimate the distribution of small water bodies for 30 m dataset, especially WBs smaller than 0.01 km^2^. The number and area of WBs larger than 0.01 km^2^ from 10m-resolution data is in agreement with that from 2m-resolution dataset. The influence of resolution on perimeter estimation is greater than that on area estimation, thus affecting the estimation of shoreline shape. Thus, the shoreline development index (SDI) increases with the increase of size of WBs. The results showed the estimation of morphological characters based on different spatial-resolution are more consistent for water bodies range from 10 to 100 km^2^. Then, the *D* of WBs in different spatial-resolution was calculated. *D* of WBs from 10 m images is 1.24 with R^2^ = 0.949, while the *D* of WBs from 30 m images is 1.36 with R^2^ = 0.919. The results indicates that the size-abundance relationships are more conform to the power law distribution when the resolution is higher. In addition, the *D* of WBs from 10-m data is close to that from 2-m data. Thus, the 10-m images could obtain approximative results with the 2-m data when only abundance estimation needed.

In the Tibetan Plateau, although small WBs are not dominant in surface, it does not preclude small WBs from significance in regional biogeochemical cycles^42^. Small WBs typically have higher fluxes and faster reaction rates than large lakes and consequently may still contribute disproportionately to biogeochemical cycles of lake-rich regions^43^. Our dataset could be valuable to fill the gap of existing water bodies map and analyze the spatial variation of water abundance and shapes, especially for small WBs.

### Supplementary information

Supplementary

## Data Availability

Codes for the dataset pre-processing are written using python, including TIFF read, morphological opening-and-closing operation, TIFF write and mosaic process. The codes are available at: https://github.com/Siyu1993/WaterPreprocessing. Then the image could be visualized in QGIS software (V3.16).
