# Supplementary material for: A high-resolution dataset of water bodies distribution over the Tibetan Plateau
Source: Sci Data. 2024 May 4;11:453. doi: 10.1038/s41597-024-03290-4 (PMC11069532; doi:10.1038/s41597-024-03290-4)
Supplement: Supplementary file 1 — Supplementary [file 41597_2024_3290_MOESM1_ESM.pdf]

A high-resolution dataset of water bodies distribution over the Tibetan Plateau

Zhengchao Chen[1&], Linan Guo[2,3&], Yanhong Wu[2,4], Bing Zhang[5,6*], Pan Chen[7], Xuan Yang[8], Jiawei Guo[2]

[1] State Key Laboratory of Remote Sensing Science, Aerospace Information Research Institute, Chinese Academy of Sciences, Beijing 100094, China
[2] International Research Center of Big Data for Sustainable Development Goals, Beijing 100094, China
[3] China University of Mining & Technology-Beijing, Beijing 100083, China
[4] Key Laboratory of Digital Earth Science, Aerospace Information Research Institute, Chinese Academy of Sciences, Beijing 100094, China
[5] Aerospace Information Research Institute, Chinese Academy of Sciences, Beijing 100094, China
[6] University of the Chinese Academy of Sciences, Beijing 100049, China
[7] Center for Geo-Spatial Information, Shenzhen Institutes of Advanced Technology, Chinese Academy of Sciences, Shenzhen 518055, China
[8] China Remote Sensing Satellite Ground Station, Aerospace Information Research Institute, Chinese Academy of Sciences, Beijing 100094, China
&These authors contributed equally to this work

Corresponding author: Bing Zhang (zb@radi.ac.cn)

Supplementary

Table S1. WB distribution and morphometric characters in Tibetan Plateau

Size (km^2)	Number of water bodies	Area (km^2)	Shoreline perimeter (km)	Average SDI
<0.01	385027	941.4	82946.3	1.292
0.01~0.1	83063	2372.3	89314.1	1.827
0.1~1	11326	2947.7	59231.8	2.901
1~10	1516	4359.3	42660.0	4.849
10~100	370	11576.7	33473.4	4.970
100~1000	88	22688.9	30281.1	6.270
>1000	6	12409.7	5427.8	5.360

Table S2. Water landscape indices in Tibetan Plateau

Size (km^2)	PD	LPI	LSI	SPLIT
total	8.40	8.03	358.59	74.60
<0.01	409.00	0.0011	675.85	205624.83
0.01~0.1	35.01	0.0042	458.43	55400.67
0.1~1	3.84	0.034	272.74	7425.81
1~10	0.35	0.23	161.53	981.89
10~100	0.03	0.86	77.78	243
100~1000	0.004	3.79	50.26	62.61
>1000	0.00048	37.09	12.18	4.37

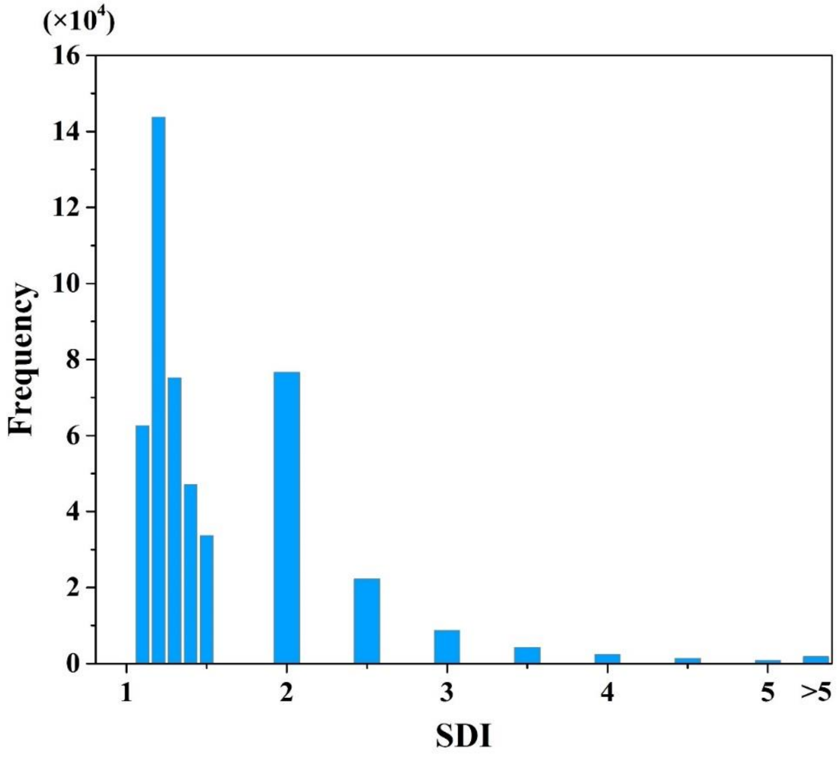

Figure S1. Histograms of Shoreline development index (SDI)
